# Culture competence and mental health across different immigrant and refugee groups

**DOI:** 10.1186/s12889-020-8398-1

**Published:** 2020-03-06

**Authors:** Brit Oppedal, Serap Keles, Charissa Cheah, Espen Røysamb

**Affiliations:** 1 Department of Child Health and Development, Norwegian Institute of Public Health, PFHI, P.O.Box 222, Nydalen, N-0213 Oslo, Norway; 2 Department of Research, The Norwegian Center for Child Behavioral Development, University of Oslo, Oslo, Norway; 3 Department of Psychology, University of Maryland, Baltimore County, Baltimore, MD 21250 USA; 4 PROMENTA Research Center, Department of Psychology, University of Oslo, Oslo, Norway

**Keywords:** Unaccompanied minor refugees, Immigrant, Youth, Young adults, Acculturation, Culture competence, Depressive symptoms, Cross-cultural equivalence

## Abstract

**Background:**

Over the last decades, due to high rates of immigration, many high-income countries have witnessed demographic shifts towards more cultural diversity in the population. Socio-economic deprivation and traumatic experiences pre-migration contribute to a high risk for mental health problems among immigrant background youth. Moreover, when adapting to the multi-cultural contexts of the resettlement countries they face several acculturation demands, which may also affect their mental health in adaptive or hazardous ways. One of these acculturation tasks involves developing the cultural competence necessary to thrive and participate socially within the heritage and the majority cultural domains. From a public mental health perspective, it is important to have thorough knowledge about acculturation-related risk and protective factors. However, this responsibility has been challenged by a lack of acculturation measures that are theoretically linked to mental health, and target the cultural competencies of immigrant background youth. Therefore, the current study aimed at examining if a construct of peer-related culture competence, operationalized in the Youth Culture Competence Scale (YCCS), captured the same competence-phenomenon across different language, age, and immigrant groups in two immigrant-receiving countries. The scale includes two dimensions: one of heritage, and one of majority peer-related culture competence.

**Methods:**

Self-report questionnaire data were collected from 895 unaccompanied refugees and 591 immigrant background high school students in Norway, and from 321 immigrant university students in the United States. To examine if the measure assessed the same phenomenon of peer-related culture competence across these three multi-ethnic samples with an age range from 13 to 28, we examined its measurement equivalence. Additionally, we examined if the association between peer-related culture competence and depressive symptoms was similar in these groups.

**Results:**

Confirmatory factor analyses supported the proposed two factor structure of the YCCS across the three samples. The structural equation model assessing the effects of heritage and majority culture competence on depressive symptoms confirmed that each culture competence dimension had a unique association with depressive symptoms across the samples.

**Conclusions:**

We conclude that the YCCS is a robust acculturation measure that may be included in public health studies of mental health among multi-ethnic refugee and immigrant samples of varied ages.

## Background

*Acculturation* is a commonly used term in scholarly discussions concerning the adaptation of immigrant and ethnic minority populations. Even if the meaning assigned to the concept differs, most researchers today agree that acculturation is a two-dimensional process that should be addressed both in relation to the heritage and the majority cultures [1, 2]. Acculturation involves a variety of individual, social, and contextual factors that may strengthen or weaken the immigrants’ mental well-being. From a public health perspective, it is therefore important to have thorough knowledge about acculturation-related risk and protective factors of mental health. A frequently researched acculturation specific risk factor is being discriminated against because of your ethnic or cultural background [3, 4]. However, less attention has been paid to acculturation specific protective factors in public health research. One reason for this may be the scarcity of acculturation measures that target the resources of immigrant background youth, and that are theoretically linked to mental health.

A major acculturation demand facing immigrant background children is to develop the cultural competence necessary to thrive and succeed within both the heritage and the majority cultural domains [5].. The Youth Culture Competence Scale (YCCS; Appendix) was developed to examine levels of such competence. As perceived competence in various domains, is a valid protective factor of children and adolescents’ mental health [6, 7], the YCCS responds to the public health sector’s need for a youth measure that is theoretically linked to mental health outcomes, and that reflects acculturation strengths. The YCCS has been employed in studies of immigrant background adolescents in Norway, and demonstrated good reliability and convergent validity in relation to depression, social support and discrimination [8–11]. However, the potential of the scale to capture the same competence phenomenon across countries, languages, age groups and voluntary and forced migrant groups, has not been assessed. Such information can add to the validity and usefulness of the scale. Therefore, the current study aimed at examining if a construct of peer-related culture competence, operationalized in the Youth Culture Competence Scale (YCCS), captured the same competence-phenomenon across different language, age, and immigrant groups in two immigrant-receiving countries. To do this, we first examined the factor structure of the YCCS in three different samples of youth in Norway and the United States to capture the culture competence phenomenon in detail. Next, we investigated the association between culture competence and depressive symptoms in these different acculturation contexts.

### The phenomenon of culture competence

The YCCS derives from an acculturation developmental psychological framework [5, 12], rather than from Berry’s model of acculturation strategies [13] to better represent the experiences of developing youths in multi-cultural contexts.

Within this framework, culture competence refers to knowledge and skills about verbal and non-verbal communication and interpersonal behavior patterns, and the values underlying these, which children develop through interaction in socio-cultural settings of both the heritage and majority culture [5, 14]. Culture competence involves both learning and maturation processes related to children’s inborn unique capacity to adapt to a variety of social and cultural circumstances, including bi- and multicultural contexts. By mentally switching between different cultural codes (scripts or schemas) they accommodate their behaviors to the demands of the context [5, 15, 16]. For children, culture competence is a necessary condition to succeed within and acquire a sense of belongingness to the two (or more) major cultural domains that constitute their developmental ecology.

The knowledge and skills involved in being culturally competent vary throughout the life span and socio-cultural domains, such as public and private domains [17]. For example, the majority culture competence (MCC) needed to participate in sports activities differs from what is needed in educational settings. Likewise, the heritage culture competence (HCC) needed to interact with family members and co-ethnic friends is also different. Moreover, the demands for MCC and HCC in interaction with friends during early and late adolescence is more complex and developmentally advanced than during the pre-school years.

It should be noted that both the definition and aim of culture competence as employed within acculturation developmental psychology differs from the use of the concept within the corporate business and health service fields. Within international business organizations culture competence describes phenomena that enable systems and professionals to work effectively in unfamiliar cross-cultural settings [18, 19]. Within health care systems the concept indicates effectively delivered health care services that meet the social, cultural, and linguistic needs of patients [20, 21].

### Measures of acculturation

There is an abundance of acculturation measures available for research, many of them thoroughly discussed by Celenk and Van de Vijver [2], Chirkov [22], and Rudmin [23]. They all advocate a two-dimensional theoretical perspectives on acculturation, i.e. assessing the individuals’ connection with both their heritage and the majority socio-cultural domains. A majority of acculturation measuring tools originated in the frequently used definition of acculturation as psychological changes that occur when people with different cultural background come into contact with each other [13, 24]. To assess such changes, these measures typically target preferences, behaviors, and activities regarding food, music, media, values, friends, and language related either to the heritage or to the majority cultural domains (for an overview and description of publicly available measures, see Celenk and Van de Vijver [2] and http://www.tilburguniversity.edu/ccis). When these measures are employed in research as predictors of mental health outcomes, the underlying assumption is that moderate to strong preference for both cultures, i.e. being bicultural, is associated with more positive mental health [13, 25–28].

From a mental health perspective, it may be argued that cultural preferences, attitudes, or strategies regarding e.g. food, music, media, values, or ethnicity of friends, which are typical indicators in measures of acculturation status, do not assess concepts with theoretical or empirical relevance as risk or protective factors of mental health. Cognitive, behavioral and interpersonal theories and research of depression and PTSD have shown that negative life events, loss, interpersonal problems, daily hassles, and traumatic exposure are major risk factors, while social support, coping, belongingness, and self-efficacy are consistent protective factors [29–37]. As can be seen, these concepts have not informed traditional acculturation measures. Thus, it is unclear how a strong preference for heritage and majority culture, as assessed by such acculturation indicators, relates to mental health. Measures that are solely rooted in theories of acculturation may not capture core aspects of the acculturation context that may increase, or protect against, the risk for mental health problems. Thus, they may be of limited value for research aiming at designing public health policies and practices to improve the mental health of immigrant background youth. For this purpose, we need an assessment tool that combines theories of acculturation with research based knowledge about risks and resources in the development of mental health problems and disorders. It is particularly valuable to identify resources, such as culture competence, that may support youth from immigrant background in handling the variety of life stressors that they are exposed to, that can be targeted in interventions to foster their resilience and positive psychological adaptation.

### The youth culture competence scale (YCCS)

Based on these considerations of the potential theoretical limitations of the existing measures, the YCCS was developed to respond to the need, from a public health perspective, for an acculturation measure that targeted children’s cultural resources, and that had conceptual links as a protective factor to depression and other indices of mental health [38, 39]. Efficacy beliefs about one’s own competence, plays a particularly influential role in human health and well-being both in terms of reducing physical stress-reactions and to facilitate the motivation and perseverance needed for success [40]. According to social-cognitive theory, self-perceived competence and efficacy are associated with self-regulatory processes, and thereby with lower levels of depressive and anxiety symptoms [6, 7, 41]. In particular, perceiving oneself to be competent in tasks that are highly valued and personally significant is strongly linked to individuals’ mental well-being, developmental psychopathology and resilience [6, 42–47]. Research has shown that competence is an important psychological construct in a variety of cultures [48].

For individuals of immigrant or refugee backgrounds, a major developmental task is the acquisition of cultural competence in the receiving-culture (MCC) and retention and further development of the heritage-culture competence (HCC). In line with two-dimensional models of acculturation, [2] the YCCS assesses these two dimensions independently. The knowledge and skills involved in being culturally competent vary throughout the life span, across socio-cultural domains, and across public and private domains [17]. For children and youth, culture competence in peer relationships is particularly important [49]. However, the demands for MCC and HCC in interaction with friends during early and late adolescence is more complex and advanced than during the pre-school years [50]. The YCCS was developed to capture the culture competence that is salient to relationships with friends during lower and upper secondary school years (ages 12 to 18 years).

Preliminary analyses of YCCS has confirmed its two latent factor structure in a multi-ethnic sample of 660 immigrant youths in Norway [51]. However, to use the scale with other samples, and in other immigrant receiving countries, we need information about its potential to capture the same competence-phenomenon across groups and context.

## Method

### Aims

The overall aim of the current study is to examine the potential of a construct of peer-related culture competence, operationalized in the YCCS, to capture the same phenomenon across different language-, age-, and immigrant groups in two immigrant-receiving countries. Importantly, by including young adult participants with age ranges from 18 to 28 years, we simultaneously assess the phenomenon of peer-related MCC and HCC in older age groups. We first examined the factor structure of the YCCS in three different samples of youth in Norway and the United States to capture the culture competence phenomenon in detail. Next, we investigated potential variation in the association between culture competence and depressive symptoms in these different acculturation groups and contexts.

### Participants

Cross-sectional data were collected from three multi-ethnic samples in the United States and Norway that comprised a total of 1807 participant. Table 1 displays the demographic characteristics for the overall sample and each subsample. The U.S. sample comprised first-generation immigrant students attending a university in the mid-Atlantic region. The Norwegian participants included one sample of unaccompanied minor refugees, and one sample of high school students with immigrant background. They all participated in The Youth, Culture and Competence Study, YCC, which had been approved by the Regional Committee for Medical and Health Research Ethics and by the Norwegian Data Inspectorate, and participation was condition on written consent. For high school students and unaccompanied refugee youth younger than 16 years, additional consent to participate in the study was collected from their parents or legal guardians respectively.

**Table 1:** Demographic characteristics of the participants (mean [SD] or %) and descriptives of the included measures, based on raw scores

Demographic Characteristics	Norway		USA	TOTAL
	Unaccompanied Refugee Sample *n* = 895	High School Sample *n* = 591	University Sample *n* = 321	*n* = 1807
Foreign born (%)				
*No*	–	57.30	–	18.20
*Yes*	100.00	42.70	100.00	81.80
Gender (%)				
Male	81.90	42.60	31.50	60.10
Female	18.10	57.40	68.50	39.90
Age				
*M*	18.62	16.75	20.89	18.41
*SD*	2.63	1.47	2.23	2.65
Length of stay (in years)				
*M*	3.44	14.36	10.41	8.17
*SD*	2.28	4.32	6.07	6.28
MCC				
*M*	2.69	3.36	3.15	3.01
*SD*	0.60	0.64	0.65	0.69
MCC-Language				
*M*	2.99	3.63	3.61	3.32
*SD*	0.64	0.61	0.65	0.70
MCC-Behavioral				
*M*	2.61	3.28	3.02	2.92
*SD*	0.69	0.71	0.72	0.77
HCC				
*M*	3.27	3.29	3.31	3.28
*SD*	0.50	0.65	0.56	0.57
HCC- Language				
*M*	3.36	2.93	3.04	3.15
*SD*	0.71	0.91	0.86	0.84
HCC- Behavioral				
*M*	3.24	3.39	3.39	3.32
*SD*	0.54	0.68	0.60	0.61
Depressive Symptoms				
*M*	20.85	16.17	17.31	18.80
*SD*	9.50	11.26	10.54	10.48

*Notes. MCC* Majority Culture Competence; *HCC* Heritage Culture Competence

#### The U.S. university student sample

The sample comprised 321 participants (*M*_age_ = 20.89 years old; *SD*_age_ = 2.23; 68.50% females) of a mid-sized state university. Students who were: (a) less than 28 years old, and (b) born outside of the United States were included in the study. Participants were predominantly juniors (34%), followed by 26% seniors, 23% freshmen, and 17% sophomores. Sixty-two percent of the participants were born in Asia, 20% were born in Africa, the rest originated from South America, Europe, and Australia. Participants had been in the U.S. for an average of 10.41 years (*SD* = 6.07).

#### The Norwegian unaccompanied refugee sample

The data were collected as part of a population-based study among youths who arrived in Norway as unaccompanied minor asylum-seekers. For detailed information about the recruitment process, see [52]. As they had received protection and consequently were no longer asylum-seekers at the time of data collection, and some of them were above the age of 18, we refer to them as unaccompanied refugees. Based on available funding and to secure demographic and socio-economic variation we targeted youth resettled in 41 municipalities nationwide, *n* = 1209, of which 948 youths agreed to participate, yielding a response rate of 78%. Thirty participants (3.2%) had missing values on most of the questions, and were therefore excluded, yielding a final sample of *n* = 918 (*M*_age_ = 18.62; *SD*_age_ = 2.63). The majority (82.1%) was male, mirroring the fact that most unaccompanied minor asylum-seekers and refugees, are boys. Their average length of stay since arrival in Norway at the time of the data collection was 3.42 years (*SD* = 2.27). Participants originated from a total of 33 different countries, mainly Afghanistan (50.8%), Somalia (12.1%), Iraq (7.1%) and Sri Lanka (6.4%).

#### The Norwegian high school sample

The sample involved 591 students with two immigrant parents with background from a Non-Western country, attending four high schools in the Norwegian capital (*M*_age_ = 16.75; *SD*_age_ = 1.47, 42.6% male). About half the participants (57.3%) were born in Norway. The average length of stay for the foreign-born students was 11.61 (*SD* = 5.17) years. The ethnic minority sample originated from more than 40 different countries. The largest groups, comprising in total 66.9% of the sample, had origins from Somalia, Pakistan, Iraq, Morocco, Sri Lanka, Afghanistan, Iran and Turkey.

### Procedure

#### The U.S. university student sample

Students were invited to participate in the study from solicitations made through their courses and posting of flyers throughout campus, as approved by the university institutional review board. Interested students were provided a link to complete an online survey hosted by Survey Monkey (http://www.surveymonkey.com). The website allowed students to provide electronic consent, and students were able to withdraw at any point without any penalty. Upon completion of the survey, which took approximately 45 min for the full battery of measures, students were prompted to submit their names to a separate email account to earn extra credit in their courses (if eligible) or be entered into a drawing for cash prizes. The information provided by students on the survey and their names submitted to the email account was not linked in order to maintain their confidentiality.

#### The Norwegian unaccompanied refugee sample

The data collection was carried out in small groups in a local community setting familiar to the youth, such as group homes or libraries. Research assistants who were trained to use questionnaire methodology in small groups administered the self-report questionnaires in Norwegian. A protocol with standardized explanations of difficult concepts and English translation of questions were available to them. Only 15% accepted the offer to use translators who could read the questions to them in their mother tongue.

#### The Norwegian high school sample

Subsequent to the high school principals’ and administration’s approval of the project, researchers from the YCC informed members of the high school student council during one of their meetings about the aims and procedures of the project. Each of the council members was given written information with invitation letters including a consent form to present to their classmates in their respective classrooms. Students who consented to participate (about 85%) responded to electronic questionnaires during two regular school classes. Research assistants trained by the YCC research team were present in the classroom during data collection to assist the students who needed help. Non-participation was mostly due to students not being present in school on the day of data collection.

### Measures

*The YCCS* includes nine items each for assessing the majority and the heritage culture competence separately (Oppedal & Idsoe, 2015; Oppedal & Toppelberg, 2016). Each dimension includes questions about language and culturally embedded patterns of behaviors. For the purpose of the present validation study, the scale was translated into English by standard back-translation procedures.

Both higher-order MCC and HCC included: 1) one first-order language competence factor with two items, such as “How easy is it for you to speak Norwegian/English?” and “How easy is it for you to write in your mother tongue?”, respectively; 2) one first-order behavioral competence factor: seven items about understanding culturally embedded patterns of behavior and interaction. Sample items for the behavioral dimension of the MCC and the HCC included “How easy is it for you to hang out with [Norwegian/European American] peers?” and “How easy is it for you to know how to behave when visiting friends and families from your culture?”, respectively. Response alternatives varied from 1 - *very difficult* to 4-*very easy*. Cronbach’s alphas in the current study varied from .80–.93 across groups.

*Depressive symptoms* were measured by the 20-item Center for Epidemiologic Studies Depression Scale for adolescents (CES-D; Radloff, 1979). CES-D asks for the frequency of symptoms over the last week and includes dimensions of depressed affect (7 items), lack of positive affect (4 items), somatic activity (7 items), and interpersonal problems (2 items). Response categories range from 0 (*rarely/never*) to 3 (*most of the time/all the time*). A previous confirmatory factor analysis supported the use of the four dimensions across gender and various cultural groups among unaccompanied refugees as well as ethnic minority and majority youth in Norway [53]. Cronbach’s alphas in the current study varied from .86–.91 across groups.

To examine correlations among all variables, we calculated a mean sum score for each culture competence dimension ranging from 1 (*perceived low culture competence*) to 4 (*perceived high culture competence*). In accordance with directions regarding the CES-D (Radloff, 1979) we calculated a sum score for symptoms of depression ranging from 0 (*no symptoms*) to 60 (*high number and frequent symptoms*). Based on findings from previous research on unaccompanied refugees [52, 54], while testing the models we controlled for gender, age, and length of stay in the respective countries.

### Statistical analyses

Conventional analyses were carried out in SPSS version 23. We used Mplus 7 (Muthén & Muthén, 2012) to examine the invariance in the factor structure of the YCCS and the association between culture competence and depressive symptoms. The robust maximum likelihood (ML) estimator, which uses the full-information maximum likelihood method (FIML), was preferred to accommodate non-normal item distributions and missing data.

Data analyses were conducted in three stages. We first tested the proposed two higher-order factor structure of the YCCS separately for the three samples by means of confirmatory factor analyses (CFA). In evaluating the models, several fit indices were consulted: the root-mean-square error of approximation (RMSEA) and the comparative fit index (CFI). West et al. (2012) suggest that CFI > .95 and RMSEA < .05 represent a well-fitting model. They also further suggest that CFI > .90 and RMSEA < .08 represent an adequately fitting model (West, Taylor, & Wu, 2012).

Second, we performed multi-group comparisons to find the extent to which the CFA model of culture competence exhibited measurement invariance across three groups. In that stage, measurement invariance across groups was tested by comparing models with: a) all parameters allowed to vary freely across groups, configural model; b) factor loadings constrained to be equal across groups, metric model; c) both factor loadings and intercepts constrained to be equal, scalar model (Little, 2013). Nested model comparisons were conducted using an adjusted χ2 difference test due to the use of the robust ML estimator (Satorra & Bentler, 2001). However, since significance testing of ∆χ2 would not be optimal due to the large sample size, we also followed the recommendation that differences in CFI values between nested models should not exceed .01 (Cheung & Rensvold, 2002; Little, 2013).

In the third stage, we assessed the unique effects of the HCC and the MCC on depressive symptoms. For this purpose, we tested a structural model across the three groups as well as for the overall sample. We also examined whether the three groups differed significantly in the effects of the two higher-order culture competence factors on depressive symptoms by testing whether a constrained model (equal paths) did not produce significantly worse model fit than an unconstrained (free paths) model (Satorra, 2000).

## Results

### Descriptive statistics

Table 1 presents demographic information for each sample, and means and standard deviations of the measures based on raw scores. The Norwegian high school sample was on average the youngest. However, as about 40% was born in Norway (2nd generation) they had on average the longest residence, as both the Norwegian unaccompanied refugee and the U.S. university samples only comprised 1st generation youth.

Table 2 shows the correlations among all the constructs. There was a small, but significant, positive correlation between MCC and HCC. Furthermore, MCC, but not HCC, correlated significantly with age and length of stay.

**Table 2:** Intercorrelations among the observed variables for the overall sample (*N* = 1807)

	1	2	3	4	5	6
1. MCC	(.92)	.27^***^	−.34^***^	.15^***^	−.11^***^	.46^***^
2. HCC		(.85)	−.21^***^	.01	.01	−.02
3. Depressive symptoms			(.89)	.00	.03	−.21^***^
4. Gender^¤^				(−)	−.02	.30^***^
5. Age					(−)	−.00
6. Length of stay						(−)

*Notes. MCC* Majority Culture Competence; *HCC* Heritage Culture Competence.

^¤^ Males were coded as 1 and females were coded as 2. ^*^p < .05, ^**^p < .01, ^****^p < .001. Cronbach alphas in the parentheses

### The measurement model

The model fit statistics for each group and for the tests of measurement invariance across groups are shown in Table 3. In order to confirm the fit of the proposed two higher-order latent factors model, we initially conducted CFA separately for each group.^1^ The two higher-order latent factor model yielded acceptable fit to the data in all three samples (Table 3).

**Table 3:** Model fit statistics for each group and for tests of measurement invariance across groups

	χ^2^ Value	χ^2^ Scale Factor	χ^2^ df	Model Comparison	∆χ^2^	∆df	CFI	∆CFI	RMSEA	RMSEA Lower CI	RMSEA Higher CI
*Each Group*											
1. Norway - Unaccompanied refugee sample	359.78	1.2184	132				.934		.045	.040	.051
2. Norway - High school sample	507.47	1.5306	132				.918		.069	.063	.076
3. USA - University sample	357.44	1.2273	132				.909		.073	.064	.082
*Across Groups*											
1. Configural Model	1247.72	1.3254	396				.921		.061	.057	.065
2. Metric Model	1322.16	1.3168	424	Model 1 vs 2	73.04^***^	28	.916	.005	.060	.057	.064
3. Scalar Model	1632.82	1.2976	456	Model 2 vs 3	362.08^***^	32	.891	.030	.067	.063	.070
3a. Partial Scalar Model	1439.84	1.3008	450	Model 2 vs 3a	126.86^***^	26	.908	.008	.061	.058	.065

*Notes.* ∆χ2 = Satorra-Bentler corrected chi-square difference test for nested models. ^*^p < .05, ^**^p < .01, ^***^p < .001

Subsequently, we tested the invariance of the measurement model across the three groups. First, the reported fit indices of a configural model in which all parameters were allowed to vary across groups, suggested an adequate fit of the two higher-order latent factor model of culture competence (see Table 3). Second, the reported fit indices of the metric invariance model in which we examined the equality of the unstandardized factor loadings across groups, also suggested an adequate fit. The CFI values differed by less than 0.01 between these two models (ΔCFI = .005), suggesting a full metric invariance implying that the indicators were related to the latent factor equivalently across the three groups. This suggests that the same latent factors were measured in each of the three groups. Third, the scalar invariance model involved examining the equality of the unstandardized indicator intercepts across groups. This invariance model showed poorer fit to the data (Table 3) and significantly worse than the metric invariance model (∆χ^2^_(9)_ = 362.08, *p* < .001, and ΔCFI>.01). The modification indices suggested freeing the intercept of indicator 8 (“How easy is it for you to enjoy yourself when spending time with [Norwegian/European American] peers”?), in addition to the factor means for the first-order MCC and HCC behavioral latent factors across groups. This partial scalar invariance model did not have a significantly worse fit than the full metric invariance model (ΔCFI = .008).

**Fig. 1:**
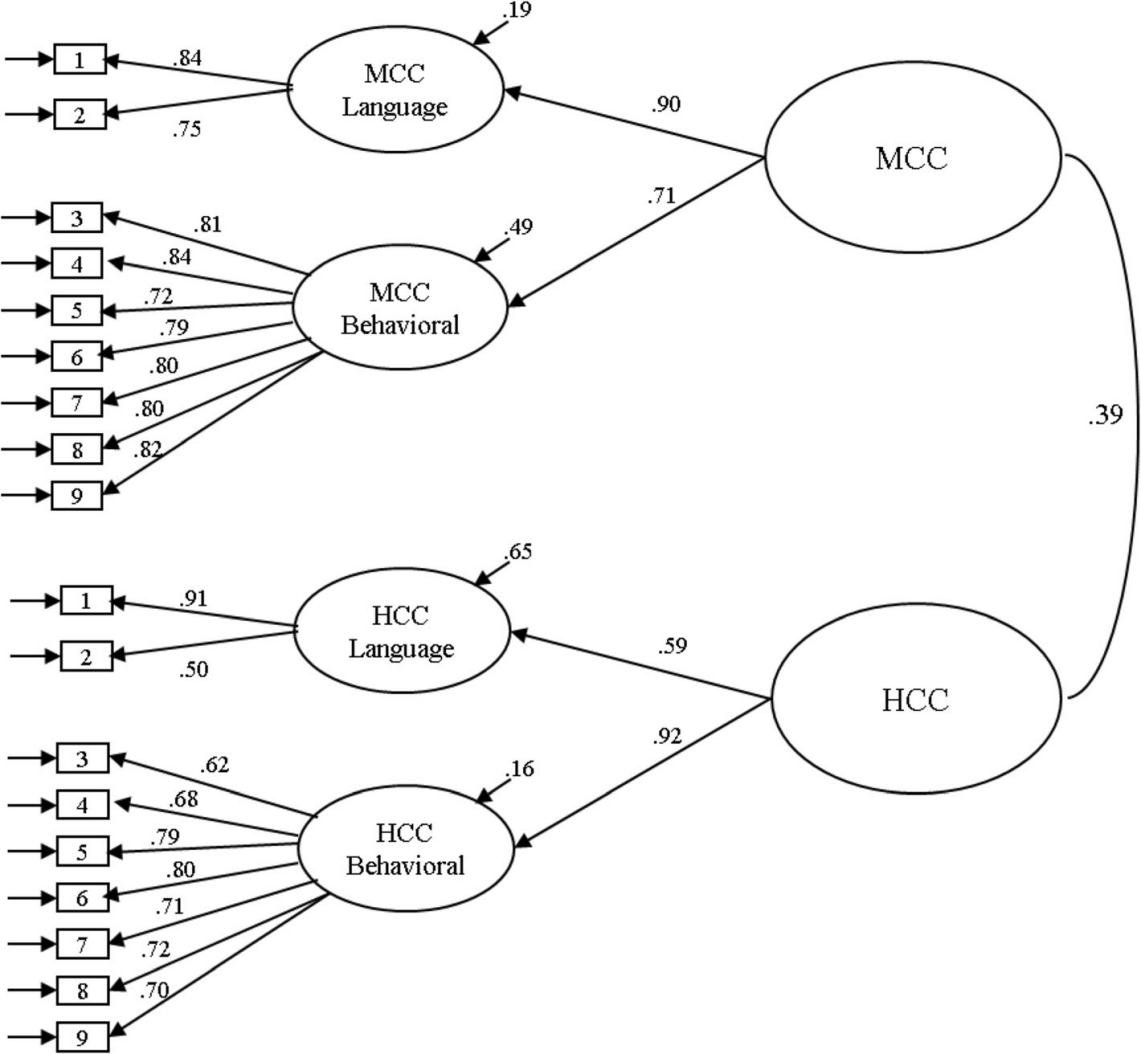
Final measurement model with two higher-order culture competence latent factors for the overall sample (*N* = 1807). Standardized parameter estimates are reported. The residual variance components (error variances) indicate the amount of unexplained variance. All factor loadings are significant at *p* < .001. MCC = Majority Culture Competence; HCC = Heritage Culture Competence

Figure 1 displays the final measurement model with two higher-order culture competence latent factors for the overall sample.

### Association between MCC-HCC and depressive symptoms

**Fig. 2:**
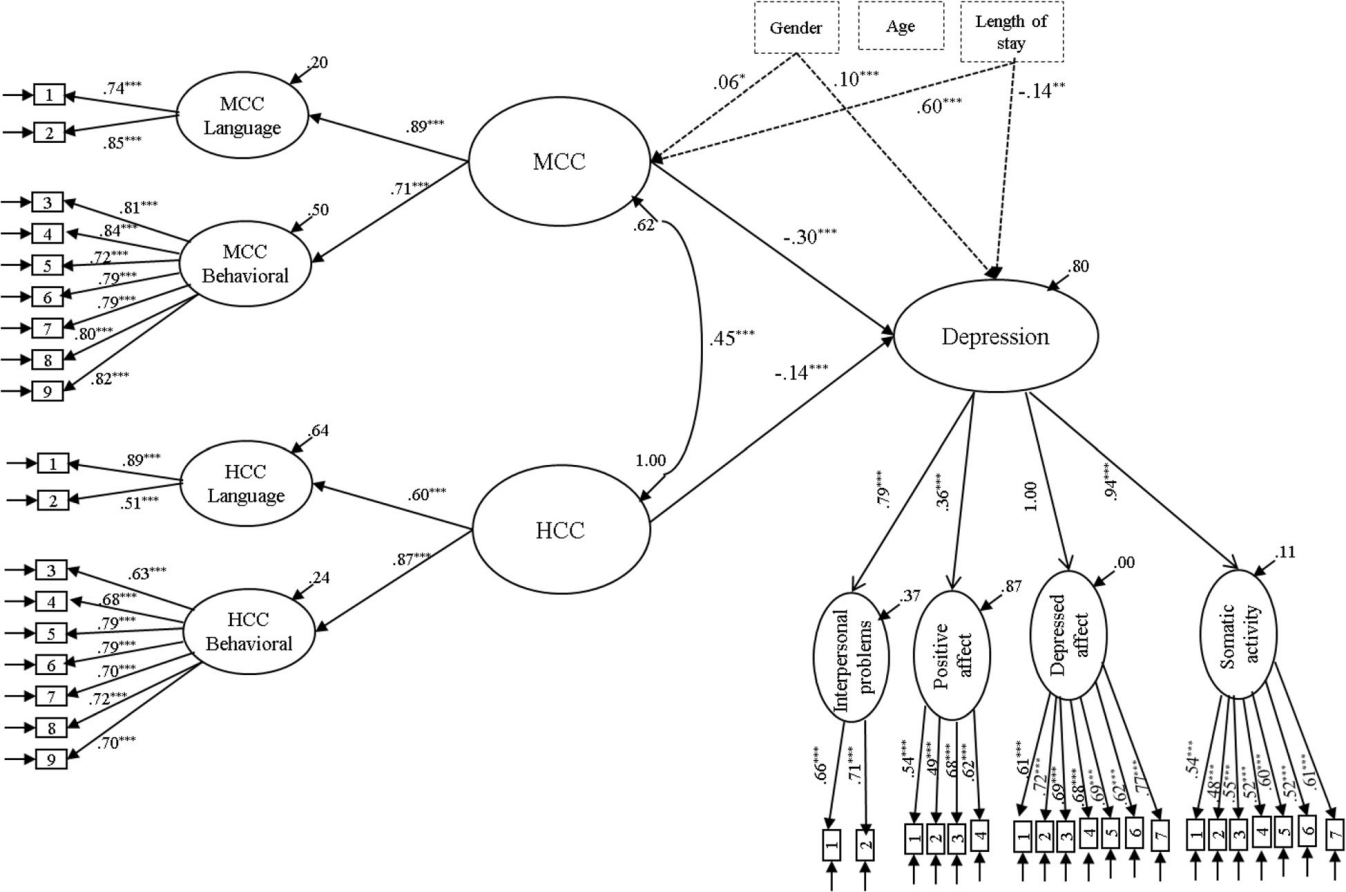
The structural model of the associations of *c*ulture competence with depressive symptoms for the overall sample (*N* = 1807). Standardized parameter estimates are reported only for the significant paths. The residual variance components (error variances) indicate the amount of unexplained variance. ^*^p < .05, ^**^p < .01, ^***^p < .001. MCC = Majority Culture Competence; HCC = Heritage Culture Competence

In order to examine the association between culture competence and depressive symptoms, we first tested a structural model in which the two higher-order MCC and HCC latent factors uniquely predicted symptoms of depression in the total sample. The structural model provided adequate fit to the data (χ^2^_762_ = 2479.99, *p* < .001, RMSEA = .036, 95%CI [0.034, 0.038], CFI = .919), and showed that higher levels of MCC and HCC predicted lower levels of depressive symptoms (Fig. 2). The subsequent multi-group structural model in which all parameters were allowed to vary across groups (free structural model) also yielded an adequate fit to the data, χ^2^_2340_ = 4360.25, *p* < .001, RMSEA = .039, 90% CI [0.037, 0.040], CFI = .904. From Table 4 it can be seen that the standardized beta coefficients from the two higher-order culture competence factors to depressive symptoms varied in size among the three subsample.

**Table 4:** Standardized beta coefficients from the two higher-order culture competence factors to depressive symptoms (the multi-group free structural model)

	Norway		USA
	Unaccompanied Refugee Sample *n* = 895	High School Sample *n* = 591	University Sample *n* = 321
MCC	−.211^***^	−.338^***^	−.163
HCC	−.166^**^	−.056	−.235^*^

*Notes. MCC* Majority Culture Competence; *HCC* Heritage Culture Competence

*p < .05, **p < .01, ***p < .001

We therefore examined whether the size of the effects of the MCC or the HCC on depressive symptoms were *significantly* different across the three groups by running two constrained structural models. Neither the first model in which the path from the MCC to depressive symptoms was constrained across groups, nor the second model in which the path from the HCC to depressive symptoms was constrained across groups, differed significantly from the free model (∆χ^2^_2_ = 1.76, *p* = .415 and ∆χ^2^_2_ = 3.83, *p* = .147, respectively). Thus, the effects of MCC and HCC on depressive symptoms were also invariant across groups.

## Discussion

This study examined if a construct of peer-related culture competence, operationalized in the Youth Culture Competence Scale (YCCS), captured the same competence-phenomenon across in three different immigrant-background groups of different ethnicities, languages and ages, in two immigrant-receiving countries. The YCCS includes two dimensions: one of heritage, and one of majority peer-related culture competence. We approached the goal by first examining and comparing the factor structure of the scale among the three groups. Next we investigated the association between MCC and HCC respectively, and depressive symptoms, in the three different samples of refugee and immigrant background youth of varying ages and ethnicities in Norway and the United States.

### YCCS factor structure invariance

Overall, the factor structure of the YCCS was supported across the three different groups of immigrant-background youth, implying that the same concepts were measured in all three samples.

The measurement model involving one language and one behavioral first order latent factor for each culture competence dimension, and two higher order latent culture competence factors had acceptable fit across the three samples. Importantly, the multi-group assessment of the factor structure showed an adequate fit for a metric model, implying that the same latent factors were measured in each of the three groups. In the partial scalar model that we preferred because it was more parsimonious than the metric model, there was variation in the intercept of the means of the latent behavioral factors of MCC and HCC. This finding implies differences among the groups in the level of culture competence, which was expected. The acquisition and maintenance of culture competence is affected by a series of individual and environmental variables such as length of stay in the receiving country, attitudes towards maintaining heritage culture, frequency of interaction with, and the supportive quality of, social networks of either culture [9].

The presence of culturally competent role models and productive feedback from significant others can also influence the development of culture competence [40]. Furthermore, the level of competence of the comparison group that is the basis for the youths’ self-evaluation may inflate or deflate their perception of their own competence [10, 55, 56].

The YCCS was primarily developed to be used with junior and senior high school students. The present validation study included participants beyond high school up to 28 years of age, and our findings support the utility of this scale in samples of young adults. Thus, based on the established partial scalar invariance of the scale in the present study, we conclude that the analyses support the generalizability of the YCCS as a measure of cultural resources to older age groups, in different migrant groups, across different acculturation contexts [57, 58]. Future research should extend on these findings and include immigrant groups in other receiving countries and non-academic contexts.

### Association of Culture Competence with depression

The final multi-group examination of a structural model involving the effects from MCC and HCC on depression symptoms supported previous findings, as both culture competence dimensions had unique effects on depressive symptoms [9, 59]. This is also in accordance with findings from other studies, showing that biculturalism is generally beneficial for the psychological adaptation of immigrants [28]. There was no significant group-variation in the size of the effects of MCC and HCC, which counters previous research with other samples in which differences in the effect sizes of MCC and HCC on depressive symptoms were found [10, 60, 61]. Notably, the Dalhaug et al. study (2011) found that the effect of MCC was non-significant in a school context involving 90% immigrant background students, whereas the effect was significant in a context with 60% immigrant background students.

Theoretical formulations and empirical findings have pointed out that competence of importance to the self, and the capacity to exercise control over things one values is critical to depression [6, 62]. Factors such as age, developmental stage, contextual demands, ratio of non-immigrant to immigrant background population in the community, and comparison group can impact the individuals’ perception of the relevance and value of specific competencies [6, 42, 63, 64]. Future experimental and non-experimental research should seek to disentangle developmental, individual and contextual predictors of culture competence and its relation with mental health indices. This may provide important insights into how children and youth learn and develop cultural resources they need to participate and adapt successfully in acculturation contexts.

### Limitations

Several limitations of the present study should be noted. First, there is an imbalance in the number of items in the verbal and non-verbal dimensions of the YCCS (i.e., two versus seven items). Including language competence items within a broader range will likely increase individual and group differences in MCC and HCC, which will contribute to improving the psychometric qualities of the measure. However, short scales are of value in psychological and health research, and the current version of the YCCS generally demonstrate good psychometric properties, and can be recommended for use with relevant populations.

Second, the information obtained was based on self-reported questionnaire information only. A mixed-methods approach to assessing cultural competence, involving additional information from tests and observations based on standardized criteria, as well as reports from friends, family, or teachers, could have added more broad and objective information. However, it should be noted that the individual’s own perceived competence within a valued domain is of most importance to his or her mental health [42].

The inclusion of, and comparison with a traditional acculturation measures would have added strength to our study. Unfortunately, this was not part of our research design. Comparison of variations in the associations with mental health between various acculturation measures, could be of value to better understand the complexities of the link between acculturation and health among immigrant background youth.

Finally, although the present study supports the use of the YCCS in acculturation contexts outside Norway and in older age-groups, more languages and variation in acculturation contexts are still needed to make final conclusions about the invariance and validity of this new acculturation measure.

## Conclusion

The results supported the importance of culture competence in relation to mental health. Furthermore, the YCCS captured a similar phenomenon of cultural competence within the peer context in three samples of refugee- and immigrant- background adolescents and young adults with different ethnic heritage backgrounds in Norway and the United States. The study also corroborates the applicability of the YCCS as a measure to assess competence relevant to youth mental health throughout the high school years and into young adulthood among university students. This implies that the YCCS is a robust acculturation measure that may be included in public health studies of mental health among multi-ethnic refugee and immigrant samples of varied ages.

## Data Availability

The raw data is confidential and cannot readily be shared. Data may be shared with researchers obtaining permissions from The Norwegian Institute of Public Health, Department of Child Health and Development and the Regional Committees for Medical and Health Research Ethics. After permissions have been obtained, data can be made available from The Norwegian Institute of Public Health, contact: Department director Heidi Aase: Heidi.aase@fhi.no.

## Appendix

**Table 5:** The youth culture competence scale

How easy is it for you ….				
	Very difficult	Somehow difficult	Somehow easy	Very easy
… to speak in English?				
… to make new friends among the European American students at school (work)?				
… to have a good time when you are with European American peers?				
… to feel you have a lot in common with peers from your own culture?				
… to make new friends from your own culture at school (work)?				
… to speak in your mother tongue? (your culture’s primary language)				
… to know how to behave when visiting European American friends and their families?				
… to enjoy yourself when spending time with peers from your own culture?				
… to feel you have a lot in common with European American peers?				
… to feel that European American peers understand you and who you are?				
… to have a good time when you are with peers from your own culture?				
… to go home with friends from your own culture after school (work)?				
… to write in English?				
… to write in your mother tongue? (your culture’s primary language)				
… to enjoy yourself when spending time with European American peers?				
… to feel that peers from your own culture understand you and who you are?				
… to go home with European American friends after school / work?				
… to know how to behave when visiting friends and families from your own culture?				

## Abbreviations

CES-D: Center for Epidemiological Studies’ Depression Scale
HCC: Heritage culture competence
MCC: Majority culture competence
YCCS: The Youth Culture Competence Scale

## Footnotes

1 In addition to all the analyses steps, to further support the use of the two higher-order structure, we also compared the proposed two higher-order factor model to the two first-order HCC and MCC factors model, without language and behavioral competence as sub-factors. This first-order model had poor model fit indices (χ^2^_134_ = 1249.21, *p* < .001, RMSEA = .069, 90% CI [0.066, 0.073], CFI = .897) and was also significantly worse than the proposed two higher-order factor structure, ∆χ^2^_(2)_ = 350.48, *p* < .001.
